# Detection of known and novel *ALK* fusion transcripts in lung cancer patients using next-generation sequencing approaches

**DOI:** 10.1038/s41598-017-12679-8

**Published:** 2017-10-02

**Authors:** Julie A. Vendrell, Sylvie Taviaux, Benoît Béganton, Sylvain Godreuil, Patricia Audran, David Grand, Estelle Clermont, Isabelle Serre, Vanessa Szablewski, Peter Coopman, Julien Mazières, Valérie Costes, Jean-Louis Pujol, Pierre Brousset, Isabelle Rouquette, Jérôme Solassol

**Affiliations:** 1 CHU Montpellier, Arnaud de Villeneuve Hospital, Department of Pathology, Montpellier, Université de Montpellier, Montpellier, France; 2 Institut de Recherche en Cancérologie de Montpellier (IRCM), INSERM U1194, Université de Montpellier, Institut du Cancer de Montpellier (ICM), Montpellier, France; 3 CHU Montpellier, Arnaud de Villeneuve Hospital, Department of Bacteriology, Université de Montpellier, Montpellier, France; 4 Institut du Cancer de Montpellier (ICM), Department of Biopathology, Montpellier, France; 5 Department of Pathology, Institut Universitaire du Cancer Toulouse Oncopole, CHU de Toulouse, Toulouse, France; 6 Thoracic Oncology Department, Larrey Hospital, University Hospital of Toulouse, Toulouse, France; 7 CHU Montpellier, Arnaud de Villeneuve Hospital, Department of Thoracic Oncology, Université de Montpellier, Montpellier, France; 8 Laboratoire d’excellence Labex TOUCAN, Toulouse, France

## Abstract

Rearrangements of the anaplastic lymphoma kinase (*ALK*) gene in non-small cell lung cancer (NSCLC) represent a novel molecular target in a small subset of tumors. Although *ALK* rearrangements are usually assessed by immunohistochemistry (IHC) and fluorescence *in situ* hybridization (FISH), molecular approaches have recently emerged as relevant alternatives in routine laboratories. Here, we evaluated the use of two different amplicon-based next-generation sequencing (NGS) methods (AmpliSeq and Archer^®^FusionPlex^®^) to detect *ALK* rearrangements, and compared these with IHC and FISH. A total of 1128 NSCLC specimens were screened using conventional analyses, and a subset of 37 (15 *ALK*-positive, and 22 *ALK*-negative) samples were selected for NGS assays. Although AmpliSeq correctly detected 25/37 (67.6%) samples, 1/37 (2.7%) and 11/37 (29.7%) specimens were discordant and uncertain, respectively, requiring further validation. In contrast, Archer^®^FusionPlex^®^ accurately classified all samples and allowed the correct identification of one rare *DCTN1-ALK* fusion, one novel *CLIP1-ALK* fusion, and one novel *GCC2-ALK* transcript. Of particular interest, two out of three patients harboring these singular rearrangements were treated with and sensitive to crizotinib. These data show that Archer^®^FusionPlex^®^ may provide an effective and accurate alternative to FISH testing for the detection of known and novel *ALK* rearrangements in clinical diagnostic settings.

## Introduction

In the past decade, the outcomes of selected subgroups of patients with non-small cell lung cancer (NSCLC) have improved considerably with the emergence of targeted therapies for management of the disease^1^. Comprehensive molecular profiling of lung adenocarcinoma has revealed a number of actionable driver alterations that are potential targets for inhibition in approximately 60% of this subtype of lung cancer^2–4^. Among the alterations, rearrangement of the anaplastic lymphoma kinase (*ALK*) gene and echinoderm microtubule-associated protein-like 4 (*EML4*) occurs in approximately 5% of lung adenocarcinomas, representing the most frequent rearrangements. Other *ALK* fusion partners have been reported, such as *KIF5B*, and *TFG* ^5^. All identified *ALK* rearrangements harbor the 5’end of the partner (including the promoter and an oligomerization domain, which is mainly a coiled-coil domain) fused to the entire ALK kinase domain, and lead to constitutive ligand-independent kinase activation. Since ALK tyrosine kinase activity is necessary for its transforming activity and oncogenicity, several ALK kinase inhibitors have been identified and successfully validated, first in preclinical models *in vitro* and *in vivo*, and then in clinical studies. The US Food and Drugs Administration (FDA) has therefore approved the use of some small molecules in advanced *ALK*-rearranged NSCLC patients. Crizotinib, a well-tolerated first generation ALK inhibitor^3,6^, has been shown to be superior to standard chemotherapy both as a first- and second-line treatment^1,7^, while second generation ALK inhibitors, such as alectinib and ceritinib, are effective not only in crizotinib-naïve patients, but also in patients with acquired resistance to crizotinib^1,8–10^.

Fluorescence *in situ* hybridization (FISH) is currently acknowledged as the “gold standard” for detection of *ALK* rearrangements. The Vysis LSI ALK Break Apart FISH Probe Kit has been approved by the FDA as a companion diagnostic test for administration of ALK inhibitors in lung cancer patients. The immunohistochemical (IHC) method, which can detect ALK protein expression independently of the underlying mechanism mediating its overexpression, is used as a pre-screening test, alongside FISH, to determine ALK status in formalin-fixed paraffin embedded (FFPE) tissue specimens. However, even though IHC is widely implemented in pathology laboratories, easy-to-use, and automatically performed, its interpretation remains difficult to standardize and time-consuming. In addition, FISH is expensive, labor intensive, requires expert pathology assessment, and is not amenable to multiplexing.

It has been recognized that the development of molecular approaches strengthens the accuracy of *ALK* fusion diagnosis, by resolving discordant or borderline cases^11–13^. Several RNA-based methods, including the nCounter assay (NanoString Technologies), reverse transcription-polymerase chain reaction (RT-PCR), multiplex RT-PCR followed by capillary electrophoresis, and RT-quantitative PCR (RT-qPCR) have demonstrated their ability^14–21^. However, some limitations prevent their full implementation in the clinical setting. They easily highlight already known fusions, but may misdiagnose new variants and fusion partners due to the low precision of the 3′/5′ imbalance value. In addition, the multiplex capabilities of some of the techniques are limited. In this context, next-generation sequencing (NGS) amplicon-based approaches have been assessed for the detection of *ALK* fusions in NSCLC patients^22–26^. Two main molecular amplicon-based NGS approaches emerged, but have not been compared to date.

Here, we evaluated two different amplicon-based NGS methods (Ampliseq and Archer^®^ FusionPlex^®^) for the detection of *ALK* fusions in order to determine the most relevant approach available for routine clinical practice in pathology laboratories. Among a set of 1128 well-characterized FFPE NSCLC specimens, 10 and 13 samples with or without *ALK* fusion, respectively, were selected for NGS testing and results were compared to IHC and FISH. Interestingly, both amplicon-based assays gave relevant results; however, only one allowed us to detect and to correctly identify the presence of two new and one rare *ALK* rearrangements.

## Results

### Specimen characteristics

A total of 1128 NSCLC specimens submitted to the University Hospitals of Montpellier or Toulouse (France) for detection of *ALK* translocations were firstly screened using IHC. The ALK IHC-positive samples (69, 6.1%) were further explored using FISH. Among them, we randomly selected 15 samples positive for *ALK* rearrangement determined by both IHC and FISH. Twenty-two *ALK*-negative samples were also selected as negative controls. We then performed two amplicon-based NGS assays: the Ion AmpliSeq RNA Lung Cancer Research Fusion Panel and the Archer^®^ FusionPlex^®^ *ALK*, *RET*, *ROS1* v2 kit.

### Fusion gene detection using the AmpliSeq kit

**Figure 1:**
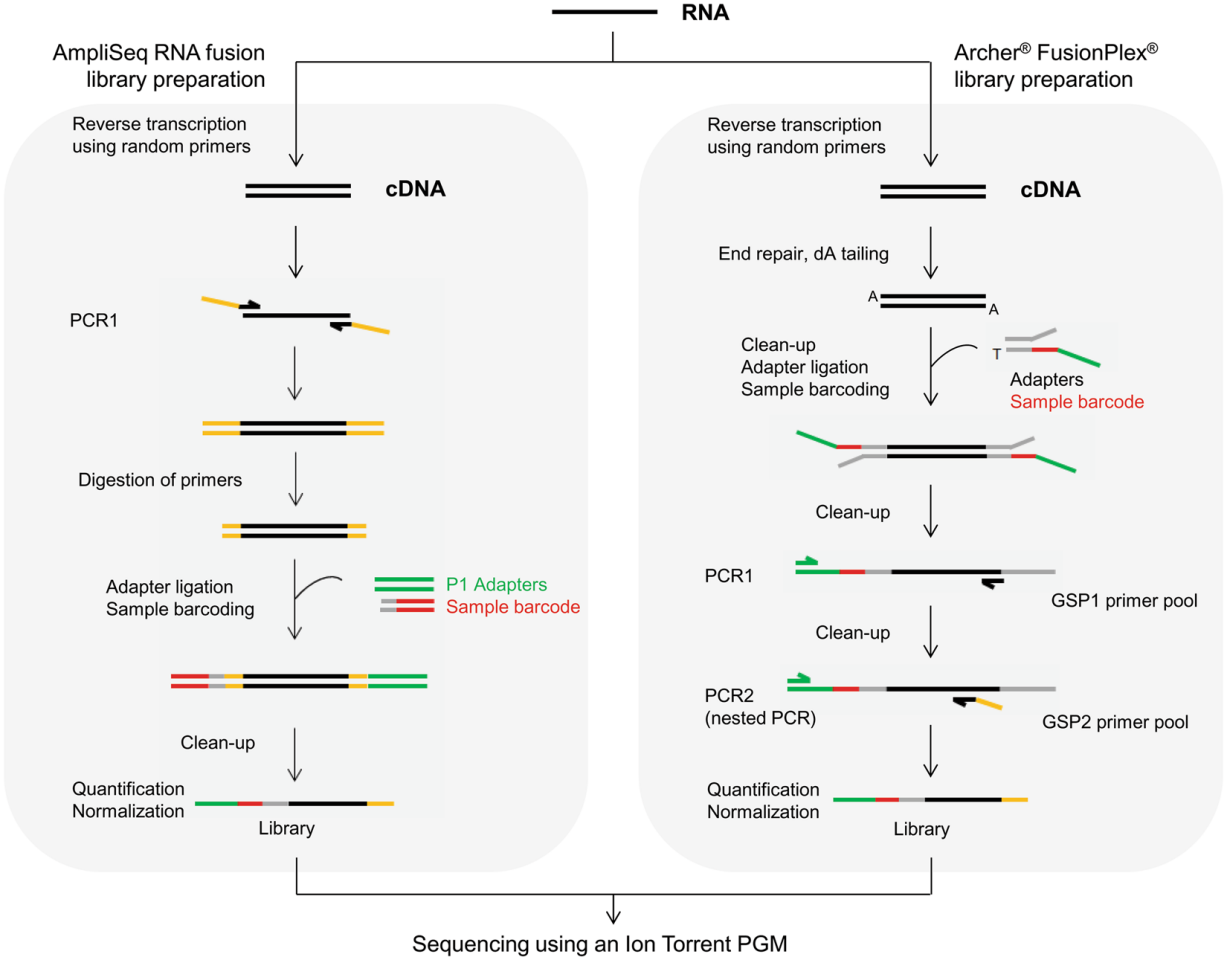
Schematic description of the library preparation workflow using the AmpliSeq RNA fusion kit or the Archer^®^ FusionPlex^®^ kit.

The Ion AmpliSeq RNA Lung Cancer Research Fusion Panel is based on an amplicon target enrichment approach that allows amplification and detection of 70 known fusion transcripts for the *ALK*, *RET*, *ROS1*, and *NTRK1* genes using a couple of primers specific of each fusion (Fig. 1). If no common fusion transcripts are detected, a 3′/5′ ratio is calculated for the four genes included in the panel and, according to the value obtained, samples are classified into three categories: no evidence, uncertain evidence, or strong evidence of the presence of a fusion. Thus, an imbalanced ratio may reflect the presence of a novel or uncommon fusion transcript in the sample that requires further exploration using a complementary molecular technique.

To assess the reliability of the panel and the inter-run reproducibility, we analyzed RNA extracted from two well-characterized control samples included in FFPE blocks in three independent experiments. These control samples corresponded to an engineered standard sample which harbored two well-characterized fusion transcripts, *EML4-ALK* (E6:A20) and *CCDC6-RET* (C1:R12), and the human lung cancer cell line H2228 (*EML4-ALK*; E6:A20). As expected, using the AmpliSeq panel for library preparations in combination with the corresponding bioinformatics analysis, the variant fusion transcripts harbored by the two control samples were correctly identified in the three independent experiments.

We next retrospectively analyzed the 37 selected tumor samples for which the *ALK* fusion status had been previously determined using conventional techniques (IHC and FISH). An *ALK* rearrangement was clearly detected in 12 cases: five *EML4-ALK* (E6:A20), six *EML4-ALK* (E13:A20), and one *EML4-ALK* (E13:A19) (Table 1). For the 25 remaining samples, as no known fusion transcript was highlighted by the analysis, the 3′/5′ imbalance values were interpreted to determine the presence or not of a potential fusion transcript (Supplementary Table S1). Among these, 13 samples did not display evidence of a fusion and were considered negative. However, for 11 other samples, uncertain evidence of an *ALK* and *RET* fusion was reported in 10 samples and one sample, respectively (Tables 1 and S1).

**Table 1:** Detection of *ALK* rearrangements in clinical specimens using IHC, FISH, AmpliSeq RNA Fusion kit, and Archer^®^ FusionPlex^®^ Kit.

Sample ID	IHC results	FISH results	NGS results		Results summary
			AmpliSeq RNA Fusion kit	Archer^®^ FusionPlex^®^ kit	
S01	Negative	Negative	Negative	Negative	Concordance
S02	Negative	Negative	Negative	Negative	Concordance
S03	Negative	Negative	Negative	Negative	Concordance
S04	Negative	Negative	Negative	Negative	Concordance
S05	Negative	Negative	Negative	Negative	Concordance
S06	Negative	Negative	Negative	Negative	Concordance
S07	Negative	Negative	Negative	Negative	Concordance
S08	Negative	Negative	Negative	Negative	Concordance
S09	Negative	Negative	Negative	Negative	Concordance
S10	Negative	Negative	Negative	Negative	Concordance
S11	Negative	Negative	Negative	Negative	Concordance
S12	Negative	Negative	Negative	Negative	Concordance
S13	Negative	Negative	Negative	Negative	Concordance
S14	Positive	Positive	*EML4-ALK* (E6:A20)	*EML4-ALK* (E6:A20)	Concordance
S15	Positive	Positive	*EML4-ALK* (E6:A20)	*EML4-ALK* (E6:A20)	Concordance
S16	Positive	Positive	*EML4-ALK* (E6:A20)	*EML4-ALK* (E6:A20)	Concordance
S17	Positive	Positive	*EML4-ALK* (E6:A20)	*EML4-ALK* (E6:A20)	Concordance
S18	Positive	Positive	*EML4-ALK* (E6:A20)	*EML4-ALK* (E6:A20)	Concordance
S19	Positive	Positive	*EML4-ALK* (E13:A20)	*EML4-ALK* (E13:A20)	Concordance
S20	Positive	Positive	*EML4-ALK* (E13:A20)	*EML4-ALK* (E13:A20)	Concordance
S21	Positive	Positive	*EML4-ALK* (E13:A20)	*EML4-ALK* (E13:A20)	Concordance
S22	Positive	Positive	*EML4-ALK* (E13:A20)	*EML4-ALK* (E13:A20)	Concordance
S23	Positive	Positive	*EML4-ALK* (E13:A20)	*EML4-ALK* (E13:A20)	Concordance
S24	Positive	Positive	*EML4-ALK* (E13:A19)	*EML4-ALK* (E13:A20)	Discordance in the variant detected by NGS approaches
S25	Positive	Positive	*EML4-ALK* (E13:A20)	*EML4-ALK* (E13:A20)	Concordance
S26	Negative	Negative	Uncertain *ALK*	Negative	Discordance between NGS results
S27	Negative	Negative	Uncertain *ALK*	Negative	Discordance between NGS results
S28	Negative	Negative	Uncertain *ALK*	Negative	Discordance between NGS results
S29	Negative	Negative	Uncertain *ALK*	Negative	Discordance between NGS results
S30	Negative	Negative	Uncertain *ALK*	Negative	Discordance between NGS results
S31	Negative	Negative	Uncertain *ALK*	Negative	Discordance between NGS results
S32	Negative	Negative	Uncertain *ALK*	Negative	Discordance between NGS results
S33	Negative	Negative	Uncertain *ALK*	Negative	Discordance between NGS results
S34	Negative	Negative	Uncertain *RET*	Negative	Discordance between NGS results
S35	Positive	Positive	Uncertain *ALK*	*GCC2-ALK* (G19:A20)	Discordance between NGS results
S36	Positive	Positive	Uncertain *ALK*	*DCTN1-ALK* (D26:A20)	Discordance between NGS results
S37	Positive	Positive	Negative	*CLIP1-ALK* (C22:A20)	Discordance between NGS results

When comparing the results obtained with the AmpliSeq RNA Fusion kit to those from the conventional techniques, concordant diagnoses were reported for 25 samples (67%) (Tables 1 and 2). Since a clear conclusion could not be made for 11 samples (30%), further experiments are required to deliver a molecular diagnosis (Tables 1 and 2). Finally, a discordant result occurred using this NGS kit for one sample (3%); an *ALK* fusion gene was detected using IHC/FISH but not using the AmpliSeq method (S37, Tables 1 and 2).

**Table 2:** Concordance between diagnoses delivered using conventional techniques (IHC and/or FISH) and NGS-based molecular approaches.

	Number of samples	NGS diagnosis	
		AmpliSeq Kit	ArcherDx Kit
Routine diagnosis (IHC and/or FISH)	25	Concordant	Concordant
	11	Uncertain^a^	Concordant
	1	Discordant	Concordant

^a^The 3′/5′ imbalance value obtained could not allow to clearly determined the presence or not of an fusion transcript in the samples. Another technique must be performed to deliver a diagnosis.

### Fusion Gene Detection in Tumor Specimens using the Archer^®^ FusionPlex^®^

The Archer^®^ FusionPlex^®^ kits are based on a different targeted enrichment method known as AMP (Fig. 1). This open-ended technique has the advantage of being able to sequence fused partners of the targeted genes without *a priori*. This permits the detection of well-described fusion events as well as previously unknown partners, with identification of the detected novel fusion transcript.

To investigate the specificity of the kit, we analyzed the RNA extracted from the two well-characterized control samples in three independent experiments. The Archer^®^ FusionPlex^®^ *ALK*, *RET*, *ROS1* v2 allowed the expected detection of the fusion breakpoints present in the samples: *EML4-ALK* (E6:A20) and *CCDC6-RET* (C1:R12) for the commercialized sample, and *EML4-ALK* (E6:A20) for the human lung cancer cell line H2228.

Using the AMP target enrichment technique on the same 37 tumor samples, we detected the presence of *ALK* fusion transcripts in 15 specimens (Tables 1 and S2). Among them, 12 harbored a common *EML4-ALK* rearrangement (E6:A20 and E13:A20). Interestingly, one novel and two rare *ALK* fusion transcripts were also identified: *GCC2-ALK* (S35), *DCTN1-ALK* (S36), and *CLIP1-ALK* (S37) (Tables 1 and S2). For 22 cases, no rearrangements were highlighted for the *ALK*, *RET*, and *ROS1* genes.

Results obtained using the Archer^®^ FusionPlex^®^ kit correlated perfectly with those from the ‘gold standard’ conventional methods. Indeed, all the specimens reported by IHC and FISH as negative cases (n = 22) or positive (n = 15) for *ALK* translocations were correctly classified using this molecular approach (Table 2). More importantly, unlike the IHC and the FISH methods, this technique allowed identification of the fusion partners without *a priori*, revealing the presence of uncommon fusion transcripts in three tumor samples in our study.

### Validation of the uncommon *ALK* fusion partners and patient clinical outcome

**Figure 2:**
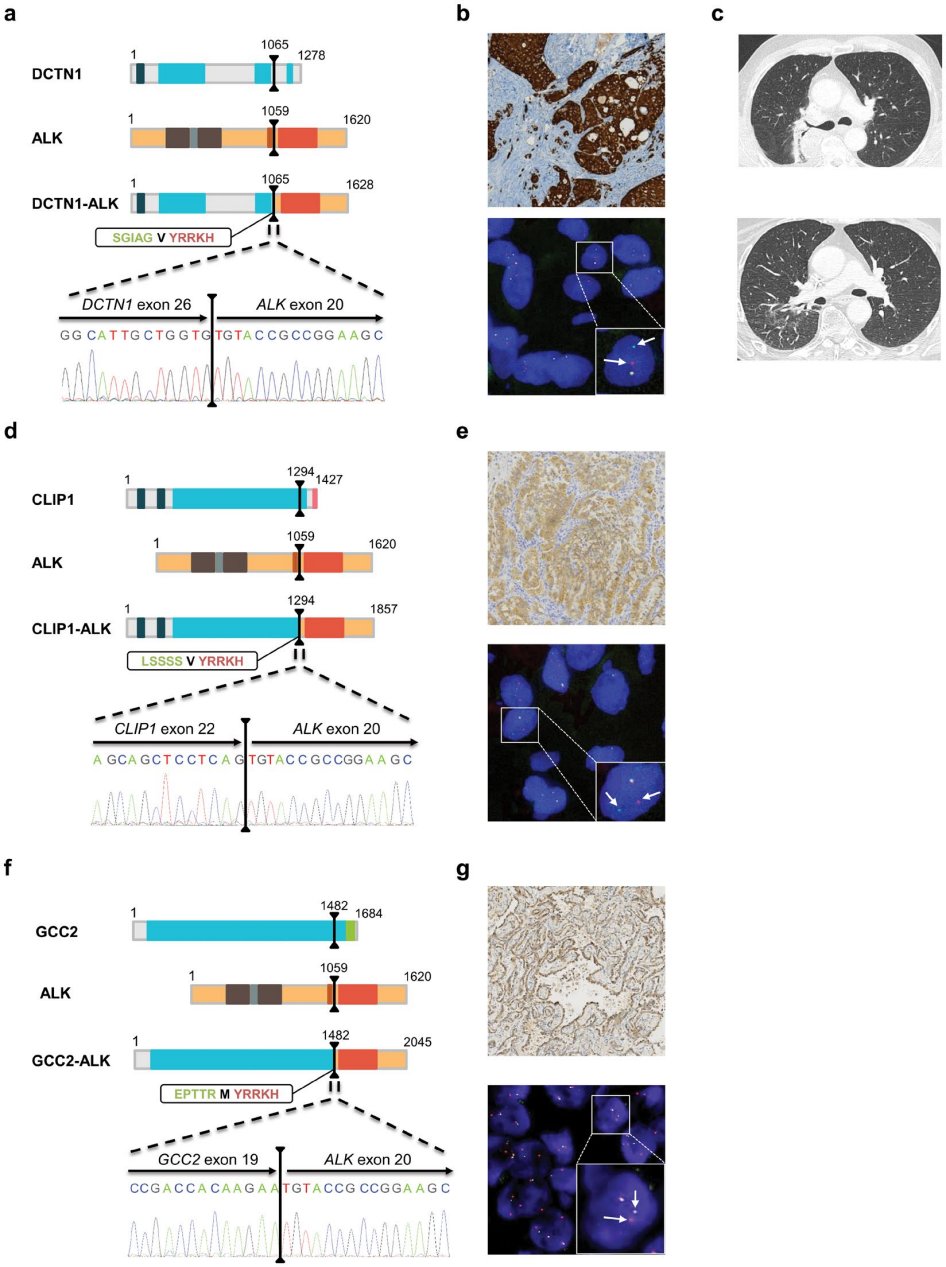
Detection of one rare *DCTN1-ALK* fusion transcript, one new *CLIP1-ALK* fusion, and one new *GCC2-ALK* rearrangement in patient samples. (**a**,**d** and **f**) Schematic representation of the main functional domains of the proteins. The black lines represent the breakpoints and the dashed lines zoom in on the transcript fusion points and the electropherogram of the validation test. The amino acid (aa) sequences at the fusion points are highlighted in a rectangle: the green bolded sequences correspond to the fusion partner, the red bolded sequence to the *ALK* sequence, and the black bolded aa to the aa generated by the fusion of the codon from the fusion partner and *ALK*. The protein functional domains are represented as colored boxes: deep blue box, Cytoskeleton-associated protein glycine-rich (CAP-Gly) domain; light blue box, coiled-coil domain; brown box, Meprin/A5-protein/PTPmu (MAM) domain; grey box, LDL-receptor class A domain; orange box, transmembrane domain; red box, kinase domain; pink box, zinc finger domain; green box, GRIP domain. (**b**,**e** and **g**) IHC and FISH images showing the presence of *ALK* rearrangements in patient samples. Top panel, IHC imaging showing an intense cytoplasmic staining. Bottom panel, representative image of a slide hybridized with a break-apart ALK FISH assay. In this given example, the box highlights one nucleus harboring a split (arrows) and a fused signal. (**c**) Thoracic CT scan of patient S36 before (top panel) and after (bottom panel) three months of crizotinib therapy. IHC, immunohistochemistry; FISH, fluorescence *in situ* hybridization; CT, computed tomography.

Among the 10 *ALK*-positive IHC/FISH samples, three exhibited a singular gene fusion detected by the Archer^®^ FusionPlex^®^ kit: *CLIP1-ALK* (S35), *DCTN1-ALK* (S36), and *GCC2-ALK* (S37) rearrangements. Primers flanking the specific fusion regions were designed and used for validation. After reverse transcription and PCR, the presence of gene fusions was further analyzed using Sanger sequencing. Of particular note, our results validated the presence of three uncommon *DCTN1-ALK* (D26:A20), *CLIP1-ALK* (C22:A20), and *GCC2-ALK* (G19:A20) fusion genes (Fig. 2). All three uncommon fusion transcripts detected included the first 1065, 1294, and 1482 amino acids (aa) of DCTN1, CLIP1, and GCC2, respectively, fused to the last 562 aa of the ALK protein and retained the intact kinase domain of ALK, which is located from 1116 to 1392 aa (Fig. 2a,d and f).

As IHC and FISH revealed an ALK-positive status (Fig. 2b), patient S36 received crizotinib orally at a dose of 250 mg twice daily in September 2015, which resulted in a significant symptomatic improvement and computed tomography (CT) response after three months of therapy (Fig. 2c). The patient remains on treatment with crizotinib and a recent CT scan demonstrated a significant shrinkage of all tumor sites outside the central nervous system. According to the ALK-positive status of patient S37 (Fig. 2e), crizotinib treatment was started in February 2016. Successive images showed a continuation of response to therapy, with stabilization of the skeletal metastases but evidence of local extension of brain metastases. Finally, despite positive IHC/FISH results (Fig. 2g), ALK-inhibitor efficiency could not be assessed in patient S35, as this patient is still in remission after surgery.

## Discussion

*ALK* gene rearrangements are usually detectable using IHC or FISH, and guide patient selection for therapy. Currently, expert consensus proposes the use of ALK IHC assays as a screening tool in two-step testing, with FISH evaluation used to validate positive or equivocal IHC samples^10,27–29^. However, several studies have reported *ALK* fusions in samples that had tested negative using IHC, demonstrating that protein expression it is not automatically linked to gene rearrangements. This highlights the risk of denying therapy with an ALK inhibitor delivery based only on IHC results^13,30–32^. In addition, the interpretation of *ALK* rearrangement by FISH strongly relies on expert experience that requires long periods of training and can be compromise by technical pitfalls^33^. Moreover, neither FISH nor IHC allow for identification of fusion partners and exact breakpoints.

Molecular diagnosis could overcome the limits of both these conventional analyses. However, to date, no technical consensus has emerged. In this study, we used two commercial amplicon-based NGS assays to determine the presence of clinically actionable *ALK* fusion transcripts. To maximize efficiency, we used 37 true NSCLC patient-derived oncology specimens previously tested for ALK by IHC and FISH. Twelve common *EML4-ALK* E13:A20 (S14–S18) and E6:A20 (S19–S25) variants were detected by both NGS approaches. The two assays displayed different results for three samples (S35, S36, and S37). The AmpliSeq amplicon-based method delivered an “uncertain” result, whereas the Archer^®^ AMP-based approach detected an *ALK*-positive fusion for all of them. Through combined RT-PCR and Sanger sequencing analysis, we validated the presence of a rearrangement in each sample. The AmpliSeq and AMP Archer^®^ FusionPlex^®^ methods identified a *EML4*(*13*)*-ALK*(*19*) fusion and a *EML4*(*13*)*-ALK*(*20*) fusion, respectively, in one FISH-positive sample (S24). This fusion was validated as *EML4*(*13*)*-ALK*(*20*) using RT-PCR and Sanger sequencing (data not shown). Importantly, among the 22 *ALK* fusion-negative samples initially detected by conventional approaches, the AMP Archer^®^ FusionPlex^®^ assay correctly established a negative result for all specimens, whereas the AmpliSeq assay was “uncertain*”* for nine of them, rendering further investigations necessary before *ALK* fusion status could be concluded^22^. Altogether, these results demonstrate the clear advantage of AMP Archer^®^ FusionPlex^®^ over AmpliSeq amplicon-based methodology in terms of giving clinically relevant, highly accurate results in a timely manner.

The Archer^®^ FusionPlex^®^ results suggest that it could be routinely used for the molecular diagnosis of NSCLC rearrangements. It is an easy-to-use laboratory test with kits developed for both PGM sequencer (Thermo Fisher Scientific) and MiSeq sequencer (Illumina) technologies. The workflow design provides a result in five working days. Furthermore, the accuracy of the test observed in our cohort demonstrates that confirmation of the result using another molecular approach is not required, as has previously been suggested^34^. There was no screening failure in our study, even though, in some cases, the RNA analyzed was extracted from samples that contained less than 20% tumor cells. In this respect, as cytological samples are the only source of material for a significant number of patients, we are planning to examine this approach in this setting. Finally, there have been concerns that the bioinformatics aspect of NGS may be challenging for regional/county hospitals. However, using Archer analysis software, we could detect and validate all known and novel rearrangements despite the absence of strong bioinformatical infrastructure in our unit, and without specific pipeline development.

Different techniques based on high-throughput molecular approaches have been improved recently, and used to detect the presence of fusion transcripts in NSCLC samples. Thus, NanoString Technologies developed a technique based on the dual hybridization of a capture probe and a molecularly barcoded reporter probe complementary to a contiguous target sequence, allowing an accurate count of molecules, even where the RNA is poor quality. The nCounter Vantage^™^ Lung Fusion Panel included junction probes specific to the fusion breakpoint, and probes upstream and downstream of a potential fusion junction for detection of gene-expression imbalance. Comparisons of NanoString performance with IHC and FISH have clearly shown showed a high degree of concordance with these gold standard techniques^17,20,35,36^. Amplicon-based NGS fusion panels have also been developed by different suppliers, and two main methods are available: the target enrichment-based (e.g. Thermo Fisher) and the AMP-based approaches (e.g. ArcherDx, Qiagen or MolecularMD). As demonstrated in this study, both molecular methods are highly sensitive, easy to perform, and give comparable results to conventional techniques. The main difference is that, unlike target enrichment-based methods, AMP-based approaches allow identification and correct naming of rare and new fusion transcripts. Indeed, although the imbalance detection used in the target enrichment-based approach is a good method to detect samples harboring new fusion genes, further experiments must then be performed to confirm the identity of the fusion partner^22,37^. Very recently, Rogers *et al*. also evaluated a new technology developed by Agena Bioscience, based on cDNA synthesis, amplification, labeling, and detection using mass spectrometry, in combination with the Agena LungFusion panel^35^. In this study, the authors compared the three transcriptome-based approaches (nCounter Vantage^™^ Lung Fusion Panel from NanoString Technologies, AmpliSeq RNA Lung fusion panel from Thermo Fisher, and Agena LungFusion panel) to FISH, and showed an overall agreement ranging from 86–96%, depending on the technique. Interestingly, both the Agena panel and AmpliSeq fusion panel reported fusions that were not detectable by FISH.

In the present study, we identified one rare *DCTN1-ALK* fusion transcript (S36), one new *CLIP1-ALK* fusion (S37), and one new *GCC2-ALK* rearrangement (S35). *DCTN1*, for dynactin subunit 1, encodes the largest subunit of dynactin, a macromolecular complex that binds to both microtubules and cytoplasmic dynein. *DCTN1-ALK* fusions have been rarely reported: four inflammatory myofibroblastic tumors (IMT)^38–40^, six Spitz tumors^41,42^, and one pancreatic tumor^43^. It has also been observed in two specimens with NSCLC^44,45^. Fusion of *ALK* with *DCTN1* induces the constitutive activation of ALK, which can be inhibited *in vitro* by treatment of the cells with crizotinib^42^. However, patients’ responses to crizotinib was poorly described in these studies and, at present, only one patient with an IMT that responded to ALK inhibitor has been reported^39^. Interestingly, the patient harboring this fusion rearrangement in our study showed sensitivity to crizotinib (S36), consistent with the results of the IMT crizotinib-treated patient.

Moreover, we highlighted, for the first time, the presence of a *CLIP1-ALK* fusion in an NSCLC sample. Although one case of a Spitz tumor harboring a *CLIP1-ALK* fusion has previously been reported, the breakpoint described differs^46^. Yeh and colleagues identified a breakpoint located between *CLIP1* exon 13 and *ALK* exon 20, whereas in our study it is between the exon 22 of *CLIP1* and 20 of *ALK*. CLIP1 protein is a member of the cytoskeleton-associated protein family with a conserved glycine-rich domain. It binds to microtubules and thereby plays an important role in intracellular vesicle trafficking. As observed for patient S36, patient S37 responded to crizotinib therapy and continues crizotinib monotherapy with no evidence of major disease progression.

Finally, we also identified, for the first time to our knowledge, a new *ALK* fusion partner: *GCC2*. The breakpoint is located between *GCC2* exon 19 and *ALK* exon 20. *GCC2*, for GRIP and coiled-coil domain containing 2, encodes for Golgi proteins involved in the tethering of transport vesicles to the *trans*-Golgi network. As previously reported for other *ALK* fusion partners, the large coiled-coil domain harbored by GCC2, when fused with ALK, may facilitate dimerization and induce the constitutive activation of ALK. The patient harboring this fusion is still in remission after surgery, rendering it impossible to determine the ALK-inhibitor efficiency in this case (S35).

The response of cell lines or patients to ALK inhibitors depending on the ALK fusion variant expressed has been insufficiently explored to date^47–49^. Of greatest interest is the observation that patients with an EML4-ALK variant 1 (E13:A20) exhibit better outcomes with crizotinib treatment than patients without this variant^49^, suggesting that ALK variants might influence the response duration of crizotinib in *ALK*-positive NSCLC. Moreover, Heuckmann and colleagues demonstrated that the cellular localization of the EML4-ALK fusion protein depends on the variant expressed, which may affect the oncogenic activity of the fusion protein^47^. At present, the specific *ALK* variant status and the fusion partner involved is not routinely considered when determining a prognosis or a therapeutic stratification for patients. Further comprehensive studies are now required to monitor patient outcomes according to the specific *ALK* variant status. AMP-based assays, which allow the precise determination of the fusion partner and breakpoint, are a simple tool for acquiring this information. This paves the way for the development of large cohort studies to determine the impact of this information on the healthcare of lung cancer patients.

With new targetable driver genes identified, and with therapeutic options evolving, a new composite decisional algorithm must be defined. As *ALK*-positive lung cancer patients benefit from tyrosine kinase inhibitor therapy in the first-line setting, *ALK* must be tested at the time of diagnosis. Our results suggest that an amplicon-based NGS assay could be performed initially. However, for laboratories that would prefer to continue using IHC as a screening test, Archer^®^ FusionPlex^®^ could be performed as a second step, to replace FISH. Moreover, since current guidelines recommend routine *ALK* testing as well as *EGFR* testing, it is important to point out that all these actionable driver genes should be tested as part of a one-test multiplex NGS panel, extracting DNA-RNA from the same FFPE sample. *ROS* and *RET* fusions, as well as a broader spectrum of genes (i.e. *KRAS*, *BRAF*, or *ERBB2*), could also be included in such routine tests. Finally, since the optimal amount of RNA recommended for Archer® FusionPlex® analysis is 200 ng (range from 20 to 250 ng), this parameter could represent a limitation for very small biopsies, even if in our study all specimens were successfully analyzed. This point has been addressed recently by Evangelista *et al*. that implemented NanoString panel for ALK fusion detection and demonstrated its applicability in series of 43 lung cancer biopsies using up to 100 ng of RNA with only 7% of sample failure^36^.

In summary, our study investigated *ALK* fusion detection based on two different commercially NGS-based approaches in FFPE-derived cancer specimens. In contrast to the AmpliSeq amplicon-based approach that was unable to detect several variants, the Archer^®^ AMP-based technique successfully identified all *ALK* fusion-positive samples, rendering this method highly applicable for routine *ALK* fusion detection and variant identification. In addition, in contrast to the conventional IHC and FISH techniques, this amplicon-based NGS approach has the distinct advantage of requiring knowledge only of one partner in the fusion. This allows the identification of novel gene rearrangements with previously unknown partners, which could clinically impact patient management.

## Materials and Methods

### Tumor samples

This study was performed with approval from the Institutional Review Board of both hospitals (Toulouse and Montpellier) and in concordance with regulatory guidelines regarding clinical assay validation. For this non-interventional study, an approved informed consent statement was acquired for all patients. FFPE tissue samples from NSCLC patients that had been submitted in 2014 to the University Hospital of Montpellier or Toulouse (France) for detection of *ALK* translocations were included in this study (n = 1128). Among them, 37 samples with previously determined *ALK* rearrangement status were randomly selected. Table 3 lists the characteristics of the patients and the corresponding specimens enrolled in the NGS assay. All lesions were submitted for pathological examination using standard techniques. The percentage of tumor cells in the specimens ranged from 10–90%. For each sample, *ALK* fusions were explored using IHC, a dual-color break-apart FISH, and NGS approaches using two different assays: Ion AmpliSeq RNA Lung Cancer Research Fusion Panel (Thermo Fisher Scientific, Waltham, MA), and Archer^®^ FusionPlex^®^ *ALK*, *RET*, *ROS1* v2 Kit (ArcherDX, Boulder, CO). Results were interpreted blindly, without knowledge of the results obtained by the other methods.

**Table 3:** Patient and specimen characteristics.

Characteristics	n	%
Sex		
Male	20	54.0
Female	17	46.0
Age		
<60	6	16.2
>60	31	83.8
Smoking status		
Have smoked	8	21.6
Smoker	9	24.3
Non-smoker	14	37.8
Unknown	6	16.2
Stage		
I	6	16.2
II	7	18.9
III	8	21.6
IV	14	37.8
Unknown	2	5.4
Type of specimen		
Biopsy	19	51.4
Surgical specimen	17	45.9
Unknown	1	2.7
Tumor cell content		
<*50*%	8	21.6
≥50%	29	78.4

### Control samples

The lung adenocarcinoma cell line NCI-H2228 (*EML4-ALK* fusion) was purchased from the American Type Culture Collection (Manassas, USA) and cultured as recommended. An FFPE Horizon Diagnostics control sample (Cambridge, UK) that harbored two well-characterized fusion transcripts was acquired (*EML4-ALK* and *CCDC6-RET* fusions).

### ALK IHC

ALK IHC was carried out using the mouse monoclonal antibody 5A4 (Abcam, Cambridge, UK) before 2015, and subsequently using the rabbit monoclonal antibody D5F3 (Roche, Basel, Switzerland). Briefly, 3–4 μm FFPE tumor tissue sections were deparaffinized and incubated in a PT link (Dako, Glostrup, Denmark) with a high pH buffer according to the manufacturer’s recommendations for antigen retrieval. Anti-ALK antibody was then applied for 30 min at 1:50. Slides were incubated at room temperature with EnVision FLEX+ Mouse Linker (Dako) for 15 min. The immune complexes were then detected with the dextran polymer reagent. The percentage of labeled tumor cells and intensity of staining were independently assessed by two pathologists.

### *ALK* FISH

Where IHC analysis was positive, FISH was performed on 3 μm FFPE tissue sections using the *ALK* FISH DNA break-apart Probe, Split Signal (Dako) according to the manufacturer’s recommendations. Slides were pretreated at 98 °C in solution for 10 min and digested with pepsin for 3 min at 37 °C using the histology FISH accessory Kit (Dako). Slides were incubated for 18 h at 45 °C with ALK probes diluted at 1:10, and had been previously denatured for 5 min at 85 °C. Slides were then washed and dehydrated before counterstaining and application of mounting medium. Slides were analyzed with a Zeiss AxioImager Z1 fluorescence microscope (Labexchange, Burladingen, Germany). Slides were analyzed independently by two pathologists. A minimum of 100 nuclei were scored and cases were considered positive when more than 15% of cells displayed split signals.

### Total RNA extraction

RNA extraction was performed on the same FFPE blocks as the IHC and/or FISH exploration. RNA was extracted from 10 µm-thick paraffin sections using the RecoverAll™ Total Nucleic Acid Isolation Kit (Thermo Fisher Scientific, Wilmington, USA) according to the manufacturer’s recommendations. RNA from control samples was extracted using the same kit. Extracted RNA was quantified using the Qubit® RNA HS Assay kit in combination with a Qubit^®^ 2.0 fluorometer (Thermo Fisher Scientific) and qualified using the RNA 6000 Nano kit in combination with the BioAnalyzer 2100™ (Agilent Technologies, Palo Alto, CA, USA). Molecular testing by the two NGS-based approaches was performed on the same RNA samples.

### Ion AmpliSeq RNA Lung Cancer Research Fusion Panel experiment

The AmpliSeq RNA Lung Cancer Research Fusion Panel is based on an amplicon sequencing approach (Table 4). The panel is composed of 83 pairs of unique primers in a single pool that includes: (i) primers that allow the amplification and detection of 70 known *ALK*, *RET*, *ROS1*, and *NTRK1* fusion transcripts; (ii) primers located in the 5′ and 3′ regions of *ALK*, *RET*, *ROS1*, and *NTRK1* mRNA genes; (iii) primers that target five housekeeping genes to serve as internal controls of the experiment.

**Table 4:** Characteristics of AmpliSeq RNA fusion and Archer^®^ FlusionPlex^®^ approaches.

Kit	AmpliSeq RNA Fusion	Archer^®^ FusionPlex^®^
Supplier	Thermo Fisher Scientific	ArcherDx
Molecular approach	Target enrichement	AMP-based method
RNA input	10 ng	200 ng
Genes in the panel	*ALK*, *RET*, *ROS*, *NTRK1*	*ALK*, *RET*, *ROS*
Panel customizable	Yes, but necessity to design a new panel as PCR primers are multiplexed together	Yes. Easy because only one primer is specific of the target region. Thus, no problem of multiplexing
Detectable alterations	Fusion transcripts	Fusion transcripts, point mutations, small insertion/deletion
Detection of new fusion variants	Yes, but only detection of the presence, not allowed the identification	Yes, with a correct naming of the breakpoint and the fusion partner
Analysis software	Provided by the supplier	Provided by the supplier
Sequencer	PGM only	PGM and Illumina sequencers

For library preparation, 10 ng of total RNA was used according to the manufacturer’s recommendations. Briefly, RNA was reverse transcribed using the SuperScript® VILO™ cDNA Synthesis Kit (Thermo Fisher Scientific). Target cDNA was amplified using AmpliSeq primer pool (Fig. 1). Primer sequences were then partially digested using FuPa reagent, and adapters and barcodes were ligated using DNA ligase. Libraries were purified with Agencourt^®^ AMPure^®^ XP (Beckman Coulter, Nyon, Switzerland), amplified by PCR as described in the user guide, purified again, and quantified using a Qubit^®^ 2.0 fluorometer using the Qubit dsDNA HS assay kit (Thermo Fisher Scientific). Based on the calculated library concentration, eight libraries were pooled to equimolar concentration. The emulsion PCR and chip loading were then performed using an Ion Chef in combination with the Ion PGM^™^ Hi-Q™ Chef kit and the Ion 318^™^ Chip kit v2 according to the manufacturer’s recommendations (Thermo Fisher Scientific). Finally, sequencing was performed on the Ion PGM sequencer using the Ion PGM^™^ Hi-Q™ Sequencing kit and analyzed by the Ion Reporter^™^ 4.4 Software (Thermo Fisher Scientific).

For samples where the software did not detect a known fusion transcript, the 3′/5′ imbalance value given by the software was used to determine the presence or not of novel or uncommon fusion transcripts^25^. For each gene present in the panel, a specific threshold has been determined by the supplier to classify samples into three categories: no evidence, uncertain evidence, or strong evidence of the presence of a fusion involving the corresponding genes.

### Archer^®^ FusionPlex^®^*ALK*, *RET*, *ROS1* v2 Kit experiment

The Archer^®^ FusionPlex^®^ *ALK*, *RET*, *ROS1* v2 kit is based on a targeted enrichment method called anchored multiplex PCR (AMP), derived from the rapid amplification of cDNA ends (RACE) method (Table 4)^50^. After reverse transcription, double-stranded cDNA undergoes end repair, adenylation, and ligation with a half-functional universal adapter (Fig. 1). Obtained cDNA are then amplified by two rounds of nested low-cycle PCR using nested gene-specific primers (GSP1 and GSP2) in combination with the first half-functional universal adapter. GSP2 primers are also 5′ tagged with a common sequencing adapter to allow the clonal amplification necessary for the sequencing step.

The panel used is composed of: (i) 29 GSP that allow the detection of gene fusion events involving *ALK*, *RET*, and *ROS*, and also *ALK* and *RET* specific point mutations, at the same time; (ii) GSP specific for five housekeeping genes.

For this kit, 200 ng of total RNA was used as input for library generation using the Archer Universal RNA reagent Kit v2, Archer Molecular Barcode (MBC) Adapters for Ion Torrent, and the Archer FusionPlex *ALK*, *RET*, *ROS* v2 Panel GSPs v2 (ArcherDX, Boulder, CO, USA) according to the manufacturer’s instructions. Briefly, RNA was reverse transcribed using random primers, first strand cDNA was synthesized, and RNA quality was assessed using the Archer PreSeq RNA QC assay (ArcherDX). After second strand cDNA synthesis, end repair and A-tailing steps were performed, cDNA was purified using Agencourt^®^ AMPure^®^ XP beads (Beckman Coulter), and MBC adapters were ligated. Purified cDNA was firstly amplified using the GSP1 pool, then purified using Agencourt^®^ AMPure^®^ XP beads, and amplified again using the GSP2 pool. After another purification step, libraries were quantified using D1000 ScreenTapes in combination with a 4200 TapeStation instrument (Agilent Technologies, Santa Clara, CA, USA) and pooled to equimolar concentration. Emulsion PCR, chip loading, and sequencing was performed as described above and results were analyzed using the Archer Analysis v3.3 software. A sample was considered as positive when the fusion breakpoint was supported by at least two unique reads.

### RT-PCR and Sanger sequencing

200 ng of RNA was reverse transcribed with random hexamers using the SuperScript^®^III First-Strand Synthesis System (Thermo Fisher). Primers specific for the detected fusion events were designed (Supplementary Table 3) and direct Sanger sequencing was performed as previously described^51^.

## Electronic supplementary material

Supplementary Tables
