# Supplementary material for: Detection of known and novel ALK fusion transcripts in lung cancer patients using next-generation sequencing approaches
Source: Sci Rep. 2017 Oct 2;7:12510. doi: 10.1038/s41598-017-12679-8 (PMC5624911; doi:10.1038/s41598-017-12679-8)
Supplement: Supplementary file 1 — Supplementary Tables [file 41598_2017_12679_MOESM1_ESM.doc]

**Detection of known and novel *ALK* fusion transcripts in lung cancer patients using next-generation sequencing approaches**

Julie A. Vendrell^1^, Sylvie Taviaux^1^, Benoît Béganton^2^, Sylvain Godreuil^3^, Patricia Audran^4^, David Grand^5^, Estelle Clermont^5^, Isabelle Serre^1^, Vanessa Szablewski^1^, Peter Coopman^2^, Julien Mazières^6^, Valérie Costes^1^, Jean-Louis Pujol^7^, Pierre Brousset^5,8^, Isabelle Rouquette^5^, Jérôme Solassol^1,2^

^1^CHU Montpellier, Arnaud de Villeneuve Hospital, Department of Pathology, Montpellier, Université de Montpellier, France

^2^Institut de Recherche en Cancérologie de Montpellier (IRCM), INSERM U1194, Université de Montpellier, Institut du Cancer de Montpellier (ICM), Montpellier, France

^3^CHU Montpellier, Arnaud de Villeneuve Hospital, Department of Bacteriology, Université de Montpellier, Montpellier, France

^4^Institut du Cancer de Montpellier (ICM), Department of Biopathology, Montpellier, France

^5^Department of Pathology, Institut Universitaire du Cancer Toulouse Oncopole, CHU de Toulouse, Toulouse, France

^6^Thoracic Oncology Department, Larrey Hospital, University Hospital of Toulouse, France

^7^CHU Montpellier, Arnaud de Villeneuve Hospital, Department of Thoracic Oncology, Université de Montpellier, Montpellier, France

^8^Laboratoire d’excellence Labex TOUCAN, Toulouse, France

Correspondence and requests for materials should be addressed to J.S. (email: [j-solassol@chu-montpellier.fr](mailto:j-solassol@chu-montpellier.fr))

**Supplementary Table 1.** 3'/5' imbalance values obtained for negative and uncertain cases using the AmpliSeq panel

^a^For samples where the Ion Reporter™ 4.4 Software (Thermo Fisher Scientific) did not detect a known fusion transcript, the 3’/5’ imbalance values given by the software were interpreted according to the following thresholds determined by the supplier.


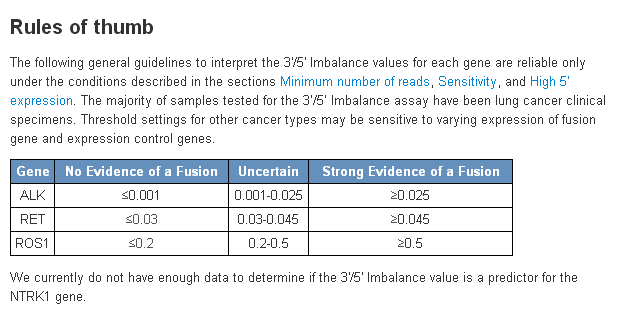


**Supplementary Table 2.** Unique fusion breakpoint spanning alignments

**Supplementary Table 3.** Primers used for RT-PCR validation
